# Experimental infection of house sparrows (*Passer domesticus*) with West Nile virus isolates of Euro-Mediterranean and North American origins

**DOI:** 10.1186/1297-9716-45-33

**Published:** 2014-03-19

**Authors:** Javier Del Amo, Francisco Llorente, Jordi Figuerola, Ramón C Soriguer, Ana M Moreno, Paolo Cordioli, Herbert Weissenböck, Miguel Ángel Jiménez-Clavero

**Affiliations:** 1 Centro de Investigación en Sanidad Animal, Instituto Nacional de Investigación y Tecnología Agraria y Alimentaria (CISA-INIA), Ctra Algete-El Casar s/n, Valdeolmos, Spain; 2 Estación Biológica de Doñana (EBD-CSIC), Avenida de Americo Vespucio s/n, Sevilla, Spain; 3 Istituto Zooprofilattico Sperimentale della Lombardia e dell’Emilia Romagna (IZSLER), Via Bianchi 7/9, Brescia, Italy; 4 Department of Pathobiology, University of Veterinary Medicine in Vienna Veterinärplatz 1, Vienna A-1210, Austria

## Abstract

West Nile virus (WNV) is a zoonotic arboviral pathogen transmitted by mosquitoes in a cycle involving wild birds as reservoir hosts. The virus has recently emerged in North America and re-emerged in Europe. North American WNV outbreaks are often accompanied by high mortality in wild birds, a feature that is uncommon in Europe. The reason for this difference is unknown, but the intrinsic virulence of the viruses circulating in each continent and/or the susceptibility to the disease of Palearctic as opposed to Nearctic wild bird species could play a role. To assess this question, experimental inoculations with four lineage 1 WNV strains, three from southern Europe (Italy/2008, Italy/2009 and Spain/2007) and one from North America (NY99) were performed on house sparrows (*Passer domesticus*), a wild passerine common in both continents. Non-significant differences which ranged from 0% to 25% were observed in mortality for the different WNV strains. Viremias lasted from 1 to 5–6 days post-inoculation (dpi) in all cases; individuals inoculated with NY99 had significantly higher titres than those inoculated with any of the Euro-Mediterranean strains. Remarkably, host competence was found to be higher for NY99 than for the other strains. Consequently, albeit being pathogenic for house sparrows, some Euro-Mediterranean strains had reduced capacity for replication in -and transmission from- this host, as compared to the NY99 strain. If applicable also to other wild bird host species, this relatively reduced transmission capacity of the Euro-Mediterranean strains could explain the lower incidence of this disease in wild birds in the Euro-Mediterranean area.

## Introduction

West Nile virus (WNV, *Flaviviridae* family, *Flavivirus* genus) is an arthropod-borne pathogen of humans, horses and some birds [1]. In recent years WNV has expanded its geographical range dramatically and is now considered to be one of the most widespread arboviruses in the world [2-4]. In North America, since its introduction in 1999, WNV has provoked thousands of cases in humans and animals, and has caused extensive mortality in wild birds [5]. By contrast, in Europe, where the disease is re-emerging, WNV only causes sporadic clinical cases and self-limited outbreaks, with no (or only very limited) wild bird mortality [4,5]. The reason for these observed differences in wild bird mortality is unknown but could be related either to the relative intrinsic virulence of the WNV strains circulating in each continent and/or to a different susceptibility to WNV infection in Palearctic as opposed to Nearctic wild bird species, given that the former have co-evolved with this pathogen while the latter are naïve in this regard.

A useful approach for investigating this issue is the performance of experimental infections in avian hosts with strains of different origins [6]. The virulence of the North American WNV prototype strain (NY99) in different avian species has been assessed experimentally in a number of studies [7-13] from which it is apparent that this strain is highly pathogenic for certain species of birds, notably – but not exclusively – Nearctic corvids [7]. The house sparrow (*Passer domesticus*) (HoSp hereafter), an abundant and ubiquitous passerine, has also been shown to be susceptible to WNV disease after experimental inoculation with the NY99 strain, with mortality rates ranging from 10% to 50% [7,14,15]. As a widespread wild bird species, HoSp could potentially represent a useful model for experimental studies on the comparative virulence of WNV strains with different geographical and/or phylogenetic origins. A recent study compared the virulence of NY99 in this host species with two other WNV strains, namely KN-3829 and Kunjin-6453 [16]. The former was isolated in Kenya in 1998 and belongs to the Western-Mediterranean-Eastern-European-Kenyan phylogenetic cluster within lineage 1a [17], while the latter was isolated in Australia in 1991 and belongs to lineage 1b. In the aforementioned study NY99 and KN-3829 induced similar mortalities, while Kunjin-6453 showed no virulence in host birds. The fact that an Old World WNV strain is as virulent as the North American prototype strain for HoSp is intriguing since it implies that the perceived mortalities in HoSp should also have occurred in the Old World WNV outbreaks when, in fact, no such observations have been reported.

Phylogenetically, KN-3829 is closely related to the majority of WNV isolates that have been circulating since 1996 in the western Mediterranean and Eastern Europe; nevertheless, despite this, 6–13 amino-acid residue substitutions still remain in the whole polyprotein sequence (3433 residues) between the Kenyan and the Euro-Mediterranean strains. Small changes in the viral RNA sequence may lead to relevant virulence shifts [18,19] and so it is possible that the observed virulence of the Kenyan strain for HoSp might not be applicable to other WNV strains belonging to the same phylogenetic cluster. In order to assess the virulence for HoSp of other WNV strains belonging to the Euro-Mediterranean cluster, wild HoSp captured in Spain were inoculated with three Euro-Mediterranean lineage 1 WNV strains, Spain/2007, Italy/2008 and Italy/2009, and the course of the infection was compared with that caused by inoculation with the NY99 strain.

## Materials and methods

### Viruses and virus preparations

The four West Nile virus strains used in this work were as follows: NY99-crow-V76/1, a North American WNV strain, isolated from a diseased crow during the WNV outbreak in New York in 1999 (GenBank accession n° FJ151394); GE-1b/B, isolated from a golden eagle that died as a consequence of WNV infection in Toledo, Spain in 2007, (GenBank accession n° FJ766331 [20]; hereafter “Spain/2007”); Italy 15803/08, isolated from a magpie hunted during a pest-control programme in Ravenna, Italy in 2008 (GenBank accession n°: FJ483549; hereafter “Italy/2008”); and Italy/2009, isolated from a yellow-legged gull found moribund in Ravenna, Italy, in 2009, along with several other WNV-infected individuals of the same species, that died afterwards in a wildlife rehabilitation centre (GenBank accession n°: JF719067). The NY99 strain was obtained through the National Veterinary Service Laboratories, USDA, lot n° 034EDV0601, while the other WNV strains were those used in our previous works [17,20,21]. All the above viruses were propagated and titrated by plaque assays in Vero cells (ATCC CCL-81) and were completely sequenced prior to inoculation in order to ascertain that the viruses inoculated had nucleotide sequences identical to those available in GenBank.

### Birds and animal care

Free-living HoSp were captured using mist nets and banded in the province of Sevilla, Spain. After habituation in captivity for two months in Sevilla, they were transported to the high-level biocontainment facility at CISA (Valdeolmos, Spain), where they were bled to determine their pre-existing immunity to WNV and housed in individual cages installed in the BSL-3 facilities. HoSp were randomly distributed in groups of 8 individuals, composed (whenever possible) of 50% males and 50% females. Mixed bird-seed and water were supplied *ad libitum*. All the animal care, handling and experimental procedures performed in this work were supervised and approved by the National Committee for Ethics and Welfare in Animal Experimentation in Spain according to European legislation (Council Directive 86/609/EEC).

### Experimental inoculations

After seven days of acclimatization in the BSL-3 room, groups of 8 HoSp were inoculated subcutaneously in the neck with 0.1 mL of viral suspension. Viruses were previously diluted in DMEM supplemented with antibiotics and glutamine, to obtain approximately 10.000 plaque-forming units (pfu)/bird of the assigned WNV strain. Each group was inoculated with a different WNV strain (see “Viruses and virus preparations” section). At the same time, a control group (*n* = 8), kept in separate cages in the same room as the infected ones, was sham-inoculated with an equivalent volume of diluent and manipulated in the same way as the virus-inoculated groups.

### Clinical follow-up and collection of samples

The experimental procedure was based on Sotelo et al. [22], albeit with some modifications. All birds were monitored daily for clinical signs of illness. To follow the course of the viremia and viral load, 0.1 mL blood samples (obtained from the jugular vein) were collected at 1, 3, 5 and 7 days post-inoculation (dpi) from half of the birds (four in each group) and at 2, 4, 6 and 8 dpi from the other half, that is, from alternating individuals in order to minimize animal manipulation, reduce unnecessary stress and limit the risk of provoking anaemia due to repeated bleeding. Similarly, oropharyngeal and cloacal swab samples were collected from all birds one day before inoculation, at 1, 5, 7 and 11 dpi from half of the birds, and at 2, 4, 8 and 12 dpi from the other half. Blood samples were collected in sterile polypropylene tubes filled with 0.9 mL BA-1 diluent (Hanks M-199 salts, 0.05 M Tris, pH 7.6, 1% bovine serum albumin, 0.35 g/L of sodium bicarbonate, 100 units/mL of penicillin, 100 μg/mL of streptomycin, 1 μg/mL of amphotericin B), mixed and stored at −70 °C until analysis. Swab samples were placed in sterile polypropylene tubes containing 1 mL PBS and immediately stored at −70 °C until analysis. Birds that succumbed to the infection were necropsied within 18 h following death. Single-use scalpels and forceps were employed during necropsy to avoid the cross-contamination of tissues. Tissue samples from the brain, kidney, heart, liver and spleen were obtained from birds succumbing to the infection, or euthanized at the end of the experiment (21 dpi). One aliquot of sample was homogenized in 0.9 mL PBS as described [22], while a second was fixed with 4% buffered formaldehyde solution and processed for immunohistochemical analysis as described [23] with the only exception that the primary antibody was from a different source (P. Emrich, Bernhard Nocht Institute, Germany). In order to obtain serum for antibody detection techniques (see below), additional blood samples (0.1–0.2 mL/individual) were taken prior to inoculation and at 14 dpi. They were collected in dry tubes and allowed to clot at 37 °C for 1 h, followed by incubation at 4 °C overnight.

### Viremia and viral load assays

Viremia was measured using a standard plaque-formation assay in Vero cells as previously described [24]. The viral load in blood and tissue samples was measured essentially as previously described [22] using a semi-quantitative real-time RT-PCR method for the detection of the lineage 1 WNV genome [25]. Virus shedding was assessed in oropharyngeal and cloacal swabs using the same procedures. A Ct = 40.0 was set as cut-off for the real-time RT-PCR used.

### Antibody detection assays

Serum antibodies to WNV were detected by a commercially available epitope-blocking ELISA (Ingezym West Nile Compac, INGENASA, Madrid, Spain) suitable for the detection of antibodies to WNV in the serum of wild birds [26].

### Calculation of host competence index (Ci) values

The calculation of competence index values for inoculated HoSp was based on the formula described elsewhere [27], considering infectious viremia values as all those above > 10^5.0^ pfu/mL for *Culex pipiens* mosquitoes as has been previously described [28].

### Statistical analysis

The viremia and blood genome load of the different inoculated groups were compared using a mixed-effects ANOVA. Individual identity was included as a random factor, while day, virus strain and the interaction between day and virus strains were included as independent factors.

## Results

### Pathogenicity and clinical signs

**Figure 1:**
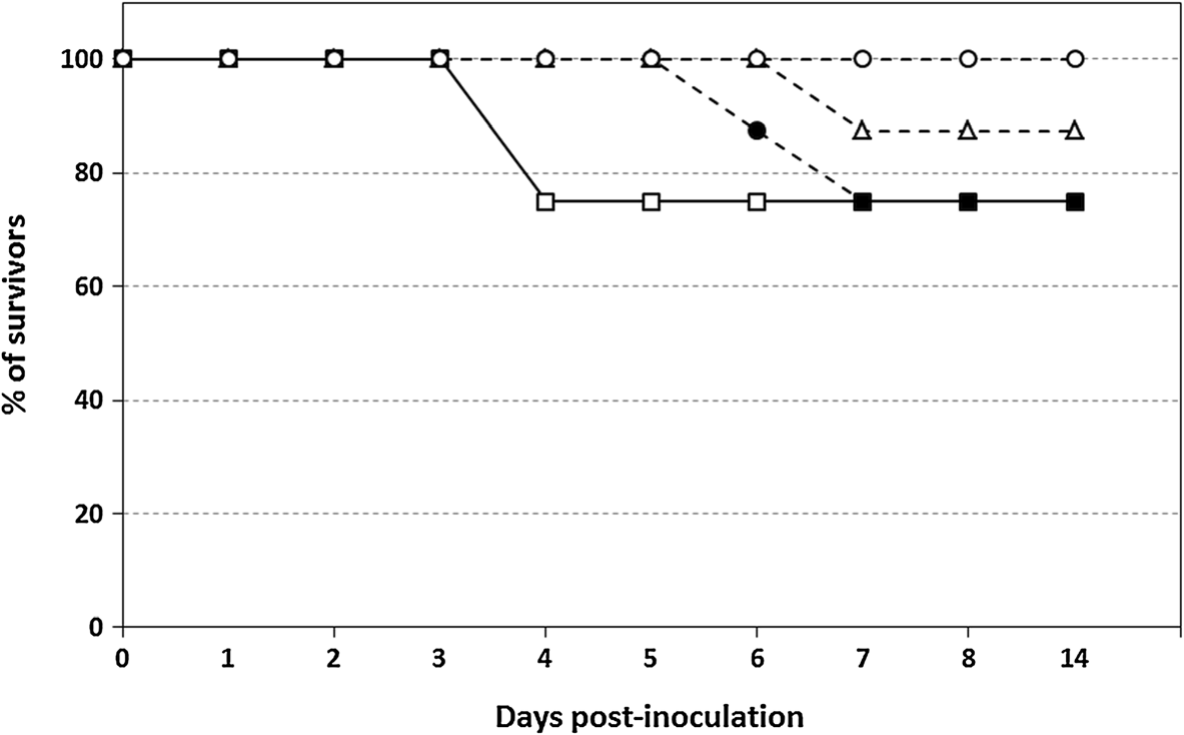
**Survival of house sparrows infected with different WNV strains.** NY99 (open squares), Spain/2007 (closed circles), Italy/2009 (open triangles) and Italy/2008 (open circles). The percentage of house sparrows surviving in each group is plotted against days post-inoculation. No mortality was found in the control group.

Table 1 provides a comparison of the deduced amino-acid sequence from each of the four WNV strains used in this work and highlights where amino-acid substitutions were found in the sequences. In the group inoculated with the NY99 strain two individuals succumbed to the infection at 4 dpi. Euro-Mediterranean strains caused either the same (Spain/2007 strain: 2/8), lower (Italy/2009: 1/8) or no mortality (Italy/2008: 0/8) (Figure 1). The deaths caused by the Euro-Mediterranean strains occurred 2–3 days (at 6–7 dpi) later than those caused by NY99 (4 dpi) (Figure 1). In addition, two sparrows in the NY99-inoculated group showed clinical signs, beginning at 4 dpi, that were characterized by abnormal posture, apathy, prostration, unresponsiveness and behavioural changes such as no avoidance of capture. These signs remained until the end of the monitoring period (14 dpi). No clinical signs were observed in any of the HoSp surviving the inoculation of the Euro-Mediterranean strains. All control sparrows survived and none were infected (not shown).

**Table 1:** Amino-acid composition of the four tested WNV strains

**Viral protein**	**Amino-acid position**	**Consensus**	**NY99**	**Spain/2007**	**Italy/2009**	**Italy/2008**
C	34	M	-	V	-	-
E	51	A	-	T	-	-
	88	P	-	S	-	-
	126	T	I	-	-	-
	159	I	V	-	-	-
NS1	35	Y	-	H	-	-
	70	S	A	-	-	-
	208	D	-	H	-	-
	284	T	-	-	M	-
	289	E	-	G	-	-
NS2A	85	I	-	-	V	V
	105	I	-	-	-	T
NS2B	103	A	V	-	-	-
NS3	249	P	-	-	T	-
	356	I	T	-	-	-
NS4A	85	I	A	V	-	-
	100	P	-	-	S	S
NS4B	115	A	-	T	-	-
	249	D	E	-	-	-
NS5	53	H	-	-	-	Y
	258	V	-	-	A	A
	422	R	-	-	K	K
	426	E	-	A	-	-
	436	M	-	I	-	-

The amino-acid sequence of the four WNV strains is compared with the WNV consensus sequence. From the left, the first and second columns display viral protein and amino-acid position, respectively, followed by the column displaying the consensus sequence and those corresponding to each WNV strain. The amino-acid residue occupying each position is indicated with the single letter amino-acid code.

### Viremia and host competence analysis

**Figure 2:**
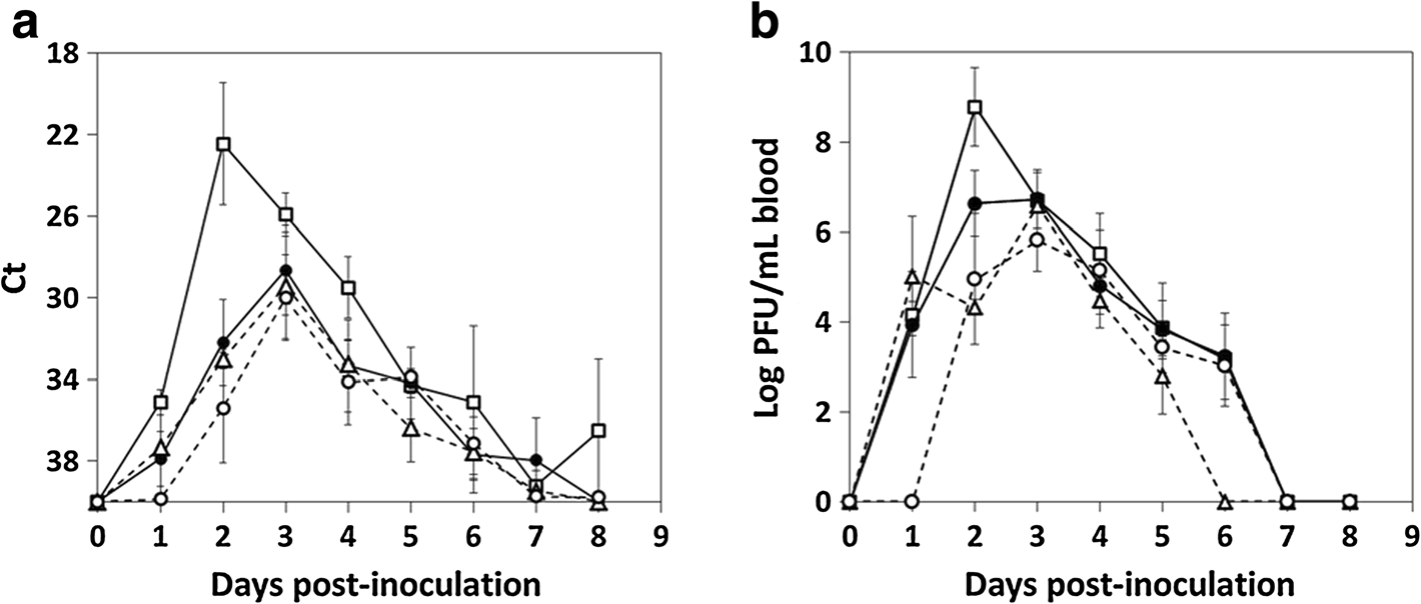
**Mean daily blood viral genome load (a) and viremia titres (b) plotted for four groups of eight house sparrows inoculated with different WNV strains.** NY99 (open squares), Spain/2007 (closed circles), Italy/2009 (open triangles) and Italy/2008 (open circles). Birds were sampled every other day as described in the text. Error bars represent the standard error of the mean.

All inoculated HoSp (except one, inoculated with Italy/2008 strain, which remained negative throughout the whole experiment) developed detectable viremias that lasted up to 5–6 dpi (Figure 2). The NY99-inoculated group showed a significantly higher mean peak viremia (*p* < 0.05) than those HoSp inoculated with the Euro-Mediterranean strains. Viral genome load data essentially corroborated the results from the viremia analyses (Figure 2). Viremias tended to be higher in the HoSp succumbing to the infection than in the survivors. This was particularly true for the two HoSp succumbing to NY99 inoculation, which reached 9.0 and 9.1 log_10_ pfu per mL of plasma respectively, while those surviving NY99 infection developed a mean peak viremia of 6.9 log_10_ pfu per mL of plasma (*n* = 6).

Based on viremia levels, it is possible to estimate the reservoir competence of a species with regards to a given viral strain [7]. A threshold of 5 log_10_ pfu/mL of blood has been calculated as the minimum amount of WNV needed to infect *Culex pipiens* mosquitoes [28]. On average, all groups of HoSp inoculated with the different WNV strains exceeded this threshold, which indicates that the HoSp is a competent host for the transmission of all the strains analyzed in this study. However, differences were observed in this respect in the competence indexes calculated for each strain (Table 2). In particular, the HoSp is a better competent host for the transmission of NY99 (Ci = 1.15) than for the Euro-Mediterranean strains that were examined (Ci = 0.17-0.47).

**Table 2:** Host competence index calculated for each group of house sparrows

**Strain**	**Susceptibility (s)**	**Infectiousness (i)**	**Mean duration (d)**	**Ci**
NY99	1.00	0.37	3.11	1.15
Spain/2007	1.00	0.19	2.49	0.47
Italy/2009	1.00	0.15	1.43	0.21
Italy/2008	0.88	0.09	2.09	0.17

The competence index (Ci) reflects the relative number of infectious mosquitoes that could be derived from feeding on a particular bird species (in this case, house sparrows) infected by a given virus strain (in this case, four WNV strains: NY99, Spain/2007; Italy/2008 and Italy/2009). Ci is determined as the product of susceptibility (proportion of exposed birds that become infected), infectiousness (proportion of vectors that become infected per day) and transmissible viremia duration (number of days a bird maintains an infectious viremia).

### Virus distribution in organs

**Figure 3:**
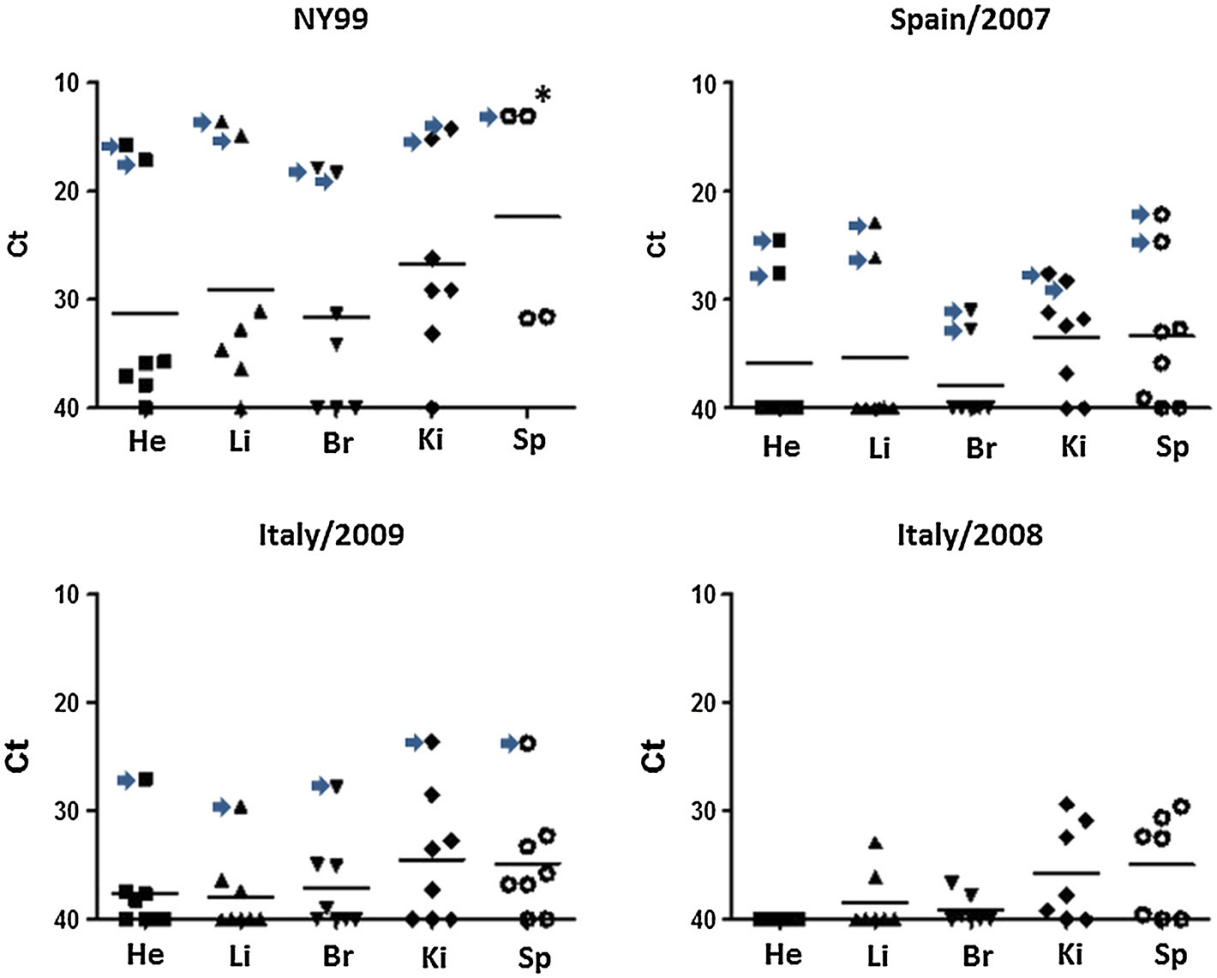
**Viral genome load in house sparrow tissues.** Viral load is plotted against each tissue analyzed by semi-quantitative real-time RT-PCR. He = heart; Li = liver; Br = brain; Ki = kidney; Sp = spleen. Each group of eight house sparrows was sampled as described in the text. Horizontal bars represent the mean Ct value of each tissue. Arrows indicate tissues from dead house sparrows. (*) only four spleens were sampled.

In all inoculated HoSp developing viremia, the infection was systemic as the virus was found in a wide range of organs upon necropsy. In general, viral loads were the highest in the spleen and then in the kidney. Liver had intermediate viral loads, while the heart and brain, the lowest values (Figure 3). Interestingly, certain differences were observed between WNV strains: in all cases HoSp inoculated with NY99 developed higher viral load in organs than those inoculated with the Euro-Mediterranean strains (Figure 3) in both dead and surviving birds (Table 3). The observed viral loads in organs from the HoSp succumbing to the infection were remarkably higher than in the organs from the surviving birds (Table 3); nevertheless, these comparisons could be misleading since survivors must have cleared most of the virus by the time they were necropsied at the end of the experiment (14 dpi). Immunohistopathological analysis of brain tissues showed WNV-specific staining only in NY99-infected HoSp (data not shown).

**Table 3:** Viral genome load in tissues from succumbing and surviving house sparrows

**Organs**	**Real-time RT-PCR results**	**Succumbing HoSp**			**Surviving HoSp (21 dpi)**			
		**NY99**	**Spain/2007**	**Italy/2009**	**NY99**	**Spain/2007**	**Italy/2009**	**Italy/2008**
Heart	Positives/total	2/2	2/2	1/1	4/6	0/6	3/7	0/8
	Ct (mean)	16.4	26.0	27.1	36.7	n.d.	37.8	n.d.
	Ct (range)	15.7-17.1	24.6-27.5	-	35.8-37.9	-	37.5-38.3	-
Liver	Positives/total	2/2	2/2	1/1	5/6	0/6	2/7	3/8
	Ct (mean)	14.2	24.5	29.6	34.5	n.d.	36.9	34.3
	Ct (range)	13.5-14.8	22.9-26.2	-	31.1-37.8	-	36.4-37.4	33.0-36.1
Brain	Positives/total	2/2	2/2	1/1	3/6	0/6	3/7	3/8
	Ct (mean)	18.0	31.9	27.9	32.3	n.d.	36.4	38.0
	Ct (range)	17.8-18.3	31.0-32.8	-	31.3-34.2	-	35.0-39.0	36.7-39.6
Kidney	Positives/total	2/2	2/2	1/1	5/6	4/6	4/7	6/8
	Ct (mean)	14.6	27.9	23.6	29.0	33.1	33.1	33.3
	Ct (range)	14.2-15.1	27.6-28.3	-	26.2-33.2	31.2-36.9	28.5-37.3	29.4-39.2
Spleen	Positives/total	2/2	2/2	1/1	2/2	4/6	5/7	6/8
	Ct (mean)	13.1	23.4	23.8	31.7	35.2	35.0	33.0
	Ct (range)	13.1-13.1	22.1-24.7	-	31.6-31.7	32.7-39.1	32.3-36.8	29.7-39.6

Viral RNA was estimated by real-time RT-PCR in tissues from five different organs of either succumbing or surviving HoSp, sacrificed 21 dpi with WNV as described in the text. Real-time RT-PCR results are expressed as positive (Ct < 40.0) over total samples analysed in each group, and as average viral genome load, expressed as mean Ct for each tissue. The Ct ranges (maximum and minimum values) obtained in each group are also indicated. (n.d.: not detectable).

### Oral and cloacal virus shedding

**Figure 4:**
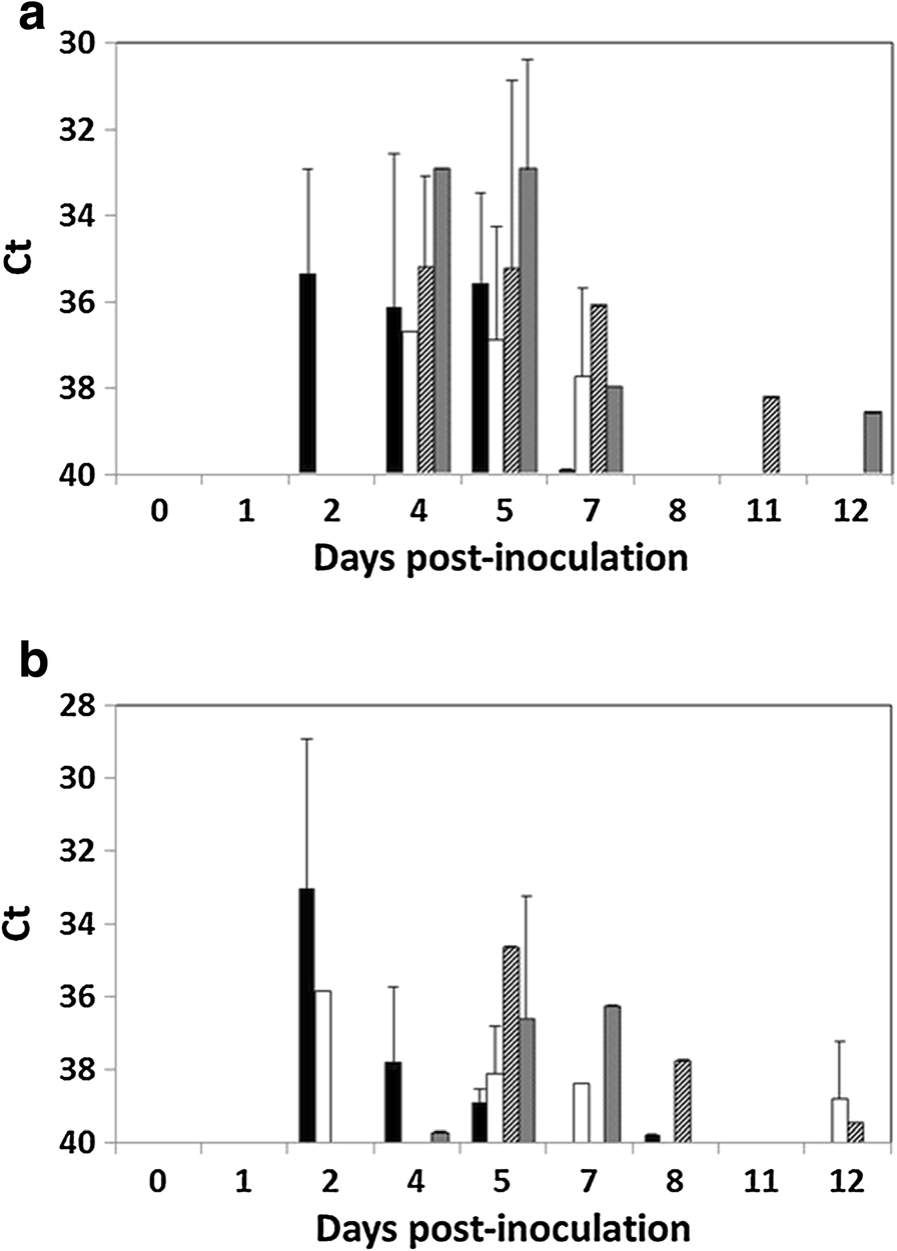
**Viral shedding through the a) oral and b) fecal routes.** Viral shedding was estimated by real-time RT-PCR analysis of the oropharyngeal and cloacal swabs taken at different days post-inoculation of WNV strains: NY99 (black bars), Spain/2007 (white bars), Italy/2009 (diagonal striped bars) and Italy/2008 (grey bars). Error bars represent the standard deviation of the means.

Virus was shed both through oropharyngeal and cloacal routes, starting from 2 dpi and lasting to 7 dpi in most cases, although some individuals still shed virus at up to 12 dpi (Figure 4). A higher proportion of individuals infected by the NY99 strain shed virus by these routes. Virus was more abundant and more consistently detected in oropharyngeal than in cloacal swabs, particularly between 2 and 7 dpi. At 5 dpi most cloacal and oropharyngeal swabs were positive. The period of oropharyngeal virus shedding for HoSp inoculated with NY99 started at 2 dpi and ceased at 5 dpi, slightly earlier than for the HoSp inoculated with the different Euro-Mediterranean strains.

### Serology

All HoSp surviving the inoculation (except the above-mentioned bird inoculated with Italy/2008 strain, which did not become viremic) developed antibodies to WNV detectable by ELISA at 14 dpi (data not shown).

## Discussion

The house sparrow is an abundant and widely distributed passerine species, which shares its habitat with humans. Although present in North America by 1850, it is in fact native to regions of Europe, Africa and Asia [29] where WNV circulates. HoSp are not only susceptible to WNV infection, but are also competent hosts for WNV transmission as has been shown in previous work [7,14,15]. However, most of these studies were performed with the North American-type strain NY99 and works of this nature using WNV strains from other continents are still few. One such study [16] revealed relatively high replication and mortality rates in HoSp caused by infection with the North American (NY99) and Kenyan (KN-3829) strains, while the Australian (Kunjin-6453) strain was found less pathogenic. In the present work, these results were extended to three new WNV strains (Spain/2007, Italy/2008 and Italy/2009) isolated in southern Europe, all of which belong to the Western-Mediterranean cluster within lineage 1a of WNV (see [17] for details of their phylogenetic assignment). The methodology used closely resembles that of the study by Langevin et al. [16] with the exception of the differing origins of the HoSp (Spain vs. US), a fact that could have a certain relevance due to possible differences in the genetic background of the birds studied. Despite this, our results confirm that HoSp is susceptible to WNV infection: while most individuals remain asymptomatic, a variable percentage suffers from more severe clinical disease that can be lethal in some instances. Thus, HoSp of European origin do not behave differently in this regard from North American HoSp. In addition, our results support the main conclusion of the study by Langevin et al. [16], that is, Old-World WNV strains can be as pathogenic for HoSp as North American ones. However, aside from the degree of virulence, we observed certain differences between NY99 and the three Euro-Mediterranean WNV strains in the course of the infection.

Overall, compared to the Euro-Mediterranean strains, NY99 produced greater viremia, earlier mortality, higher morbidity, higher viral load in organs of birds either succumbing or surviving to the infection, and earlier and more consistent virus shedding either through oropharyngeal or cloacal routes. The host-competence index calculated for NY99 was also higher than for any of the three Euro-Mediterranean strains examined, supporting the hypothesis that mosquito vectors may acquire the infection more readily when feeding from NY99-infected HoSp than from HoSp infected with the Euro-Mediterranean strains tested. It is remarkable that there are so few amino-acid changes throughout the complete polyprotein sequences (3433 amino acids) of the three Euro-Mediterranean strains examined in this work: 16 amino-acid positions differ between Spain/2007 and both Italian isolates, Italy/2008 and Italy/2009, while these latter two differ mutually in only four positions. One of these changes affects the NS3_249_ position. It has been shown experimentally that, when this position is occupied by proline (Pro) instead of threonine (Thr), the virulence of WNV for American crows (*Corvus brachyrhynchos*) is enhanced [30]. This position is occupied by Pro in NY99, Spain/2007 and Italy/2008, and by Thr in Italy/2009 (Table 1). Therefore, the mere presence of Pro at NS3_249_ was not sufficient to determine the virulence observed for HoSp in this study. This result is not surprising given that strains NY99 (NS3_249_-Pro) and KN-3829 (NS3_249_-Thr) behaved similarly in terms of virulence for HoSp [16], a finding that agrees with our previous observations [20,22].

The results shown in this work indicate that at least some WNV strains circulating in southern Europe are pathogenic for an avian species as common as the HoSp. Therefore, a certain level of mortality in HoSp should also be expected in the Euro-Mediterranean region in areas affected by WNV outbreaks. Remarkably, no substantial wild bird mortalities have ever been observed in association with WNV circulation in this region, a circumstance that contrasts with the situation in North America, where wild bird mortalities (affecting HoSp among other species) are widespread [31]. Moreover, wild-bird mortality events in North America are considered as an early-warning signal for monitoring WNV circulation in the field. This type of alert based on wild-bird mortality is seldom of any – or no – use in Europe and the Mediterranean, which emphasizes that, despite dealing with the same pathogen, surveillance systems must be adapted to the peculiarities of each transmission scenario.

The difference between North America and the Euro-Mediterranean region in observed wild bird mortality due to WNV still demands a satisfactory explanation. Possible reasons including either the greater virulence of North American WNV strains or a higher susceptibility of North American bird species have been proposed. However, the results obtained in this work do not match particularly well either of these hypotheses: on the one hand, HoSp from both continents are susceptible to lethal WNV infection but, on the other, some WNV strains in Europe are not so different from the North American type strain (NY99) in terms of mortality for HoSp. Therefore, other hypotheses must be formulated and experimentally tested to explain these facts. In this regard, NY99 exhibited a higher replication capacity in HoSp than the Euro-Mediterranean WNV strains analysed. Accordingly, NY99 also had greater viremia profiles, greater virus shedding, an earlier onset of clinical signs and greater morbidity in two clinically affected individuals – neither of which recovered within the monitoring period – that under natural conditions (i.e. in the field) would have been unlikely to survive. Although this higher replication capacity did not result in a significant increase in mortality, it did give rise to higher mean peak viremias, which in turn can influence the host competence index. This index was also found to be higher for NY99 than for the other WNV strains tested. This fact reveals an enhanced amplification capacity that would hypothetically lead to more transmission events in cases of HoSp infection with NY99-like strains, eventually giving rise to a more exacerbated cycle, a greater basic reproductive ratio (R_0_), and consequently, more infections and thus more deaths. The higher viral loads observed in organs also suggest a higher potential for viremia recrudescence and long-lasting infections for NY99, thus transmission by predation would also be more likely.

The NY99, Spain/2007 and Italy/2009 strains were isolated from diseased birds in the context of WNV outbreaks (that is, accompanied by similar birds showing evidence of WNV infection that also succumbed to it), whereas the Italy/2008 strain was isolated from a magpie that was shot during a pest-control programme. Interestingly, Italy/2008 did not cause any mortality in the infected HoSp. This fact could be indicative of the potential circulation in Europe of low-pathogenicity WNV strains that could be more difficult to detect and isolate given that they are not associated with bird mortalities [32]. However, this result should not be over-interpreted since 1) the observed differences in mortality in HoSp inoculated with the different WNV strains derive from a small number of inoculated birds; 2) a given WNV strain can be highly virulent in a given host species but be of low virulence in other(s), and 3) the fact that the magpie from which Italy/2008 was isolated was shot does not strictly mean that it was not affected by the disease. Further work is necessary to determine whether or not these strains are different in terms of their virulence for a wider range of hosts.

### Conclusion

The house sparrow is a suitable model for studying West Nile virus infection. This study confirms that it is susceptible to WNV infection and disease, and is a competent host for the transmission of WNV strains from both North America and the Euro-Mediterranean region. Although the differences observed in wild bird mortality between the continents remain unexplained, variations in host competence among WNV strains circulating in each continent could play a role. Further studies are needed to clarify this finding and, in particular, more experimental infections using a wider range of WNV strains and host species from the two continents should be performed to allow for more thorough comparisons.

## Competing interests

The authors declare that they have no competing interests.

## Authors’ contributions

JA: prepared the inocula, carried out experimental infections, the clinical and analytical follow-ups, the analysis of clinical samples and the interpretation of the results, and contributed to the drafting of the manuscript; FL: prepared the inocula and carried out the experimental infections, the clinical and analytical follow-ups, the analysis of clinical samples and the interpretation of the results; JF: captured and cared for the house sparrows used in the experiment before inoculation, and collaborated in the study design, the statistical analyses and the interpretation of the results, and contributed to the drafting of the manuscript; RCS: captured and cared for the house sparrows used in the experiment before inoculation, collaborated in the design of the study and interpretation of the results, and contributed to the drafting of the manuscript; AM: isolated and prepared some of the viral stocks used in the inoculations, participated in the design of the study and in the interpretation of the results, and contributed to the drafting of the manuscript; PC: isolated and prepared some of the viral stocks used in the inoculations, participated in the design of the study and in the interpretation of the results, and contributed to the drafting of the manuscript; HW: performed and interpreted the results of the immunohistological analyses and contributed to the drafting of the manuscript; MAJC: designed and coordinated the study, participated in the experimental infections and interpretation of the results, and drafted the manuscript. All authors read and approved the final manuscript.
